# Dynamic changes of flavor characterization of lager beer during barrel aging process based on untargeted volatilomics

**DOI:** 10.1016/j.fochx.2026.103901

**Published:** 2026-04-22

**Authors:** Yaqi Shi, Junhong Yu, Lei Xing, Shumin Hu, Hualei Chen, Peng Yan, Shuping Shao, Haixin Liu, Yajie Tang, Zhaoxia Yang, Hua Yin

**Affiliations:** a Shandong Provincial Key Laboratory of Food Biological Fermentation (in preparation), Tsingtao Brewery Co., Ltd, China; b State Key Laboratory of Microbial Technology, Shandong University, Qingdao 266237, China

**Keywords:** Barrel aging, Beer, Untargeted analysis, Volatile metabolomics, Dynamic changes, Aroma profile

## Abstract

Oak barrel aging induces complex, time-dependent transformations in beer flavor. This study systematically characterized these changes through a 300-day aging experiment integrating quantitative sensory analysis, physicochemical monitoring, and HS-SPME-GC–MS-based volatile metabolomics. Barrel aging introduced distinct coconut and vanillin notes, with extended aging further intensifying woody, sweet, and caramel characteristics, while wine-like notes remained stable. Volatile profiling identified 162 significantly differential metabolites across 12 chemical classes, including esters, aromatic compounds, ketones, and heterocyclic compounds. Mantel correlation analysis revealed that three wood-derived volatiles—(*Z*)-oak-lactone, ethyl 2-furoate, and (2,2-diethoxyethyl) benzene—were strongly correlated with aging time (|r| > 0.8) and served as potential biomarkers. Notably, (*Z*)-oak-lactone was quantitatively linked to both “woody” and “sweet” odor notes (*r* > 0.89). These findings establish a predictive chemical-sensory framework for understanding flavor development during barrel aging.

## Highlights

• 300-day study maps dynamic sensory-chemical changes in barrel-aged beer.
• Integrated sensory, physicochemical, and volatilomics analyses performed.
• (*Z*)-Oak-lactone, ethyl 2-furoate, and (2,2-diethoxyethyl)benzene are aging biomarkers.
• Strong links established between specific volatile compounds and sensory attributes.
• Findings provide scientific basis for predicting beer flavor development.

## Graphical abstract

**Figure:**
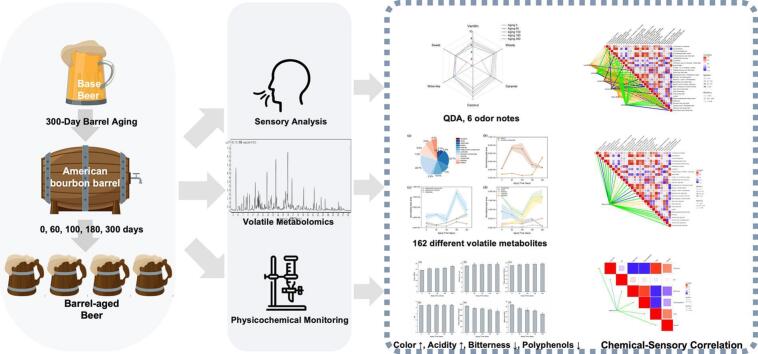

## 1 Introduction

Prior to the widespread adoption of stainless steel, wooden barrels were the primary vessels for the storage and transportation of alcoholic beverages. This tradition persists in the production of many specialty beers, wines, and spirits due to its profound impact on product character (Sterckx et al., 2012a; Tarko et al., 2023). In contemporary brewing, barrel aging is deliberately employed to design complex sensory profiles. It modulates volatile compositions to enhance aroma complexity and meet evolving consumer preferences for unique flavor experiences (Sterckx et al., 2012b). The process induces noticeable transformations in beer aroma, color, taste, and mouthfeel. For instance, French oak is known to impart vanilla and smoky notes, while American oak tends to yield a more balanced profile, better preserving the base beer's original attributes (Silvello et al., 2020; Wyler et al., 2015).

These multidimensional sensory changes are driven by intricate physicochemical interactions between the beer, the wood, and the barrel microenvironment. The process is multifaceted: solvent extraction releases wood-derived compounds such as volatile phenols, lactones (e.g., oak lactones), furans, and aldehydes, which directly contribute toasted, spicy, and vanilla notes (Coelho et al., 2019; Lu et al., 2024). Conversely, the porous wood matrix can adsorb certain beer constituents like esters and terpenes, thereby altering the beverage's native volatile profile (Lu et al., 2024). The use of previously seasoned barrels (e.g., ex-bourbon casks) introduces another layer of complexity through the transfer of residual compounds (Bossaert et al., 2021). Concurrently, microbial activity within the wood can generate organic acids and phenols, adding to flavor complexity (Bossaert et al., 2021). Underlying these physical exchanges, a suite of chemical reactions—including oxidation, hydrolysis, and esterification—occurs between beer components, wood extracts, and microbial metabolites, continuously reshaping the final aroma profile throughout maturation (Lu et al., 2024; Tarko et al., 2023). Consequently, the chemical composition of beer undergoes a dynamic and complex evolution during barrel aging, ultimately coalescing into its distinctive sensory signature. Therefore, A detailed investigation into the temporal dynamics of these chemical changes is therefore essential.

Advancements in analytical methodologies, particularly metabolomics, now enable such detailed investigations. Metabolomics provides a powerful, holistic approach for studying food composition and quality parameters like taste and aroma (Carisma & Calingacion, 2025). Its successful application in analyzing complex matrices such as vinegar, coffee, wine, whisky, and Baijiu and sake underscores its potential for deciphering the intricate chemistry of alcoholic beverages (Chen et al., 2024; Li et al., 2025; Okolo et al., 2023; Pan et al., 2022; Yu et al., 2025; Zhang et al., 2025). For craft beers, especially those undergoing transformative processes like barrel aging, metabolomics is a promising tool for uncovering the chemical phenomena underlying sensory differences and identifying potential biomarkers related to production processes.

Preliminary studies have established a foundational understanding of the chemical and sensory shifts induced by barrel aging. Our previous work confirmed that barrel aging significantly alters the aroma profile of beer, introducing notes such as “coconut”, “woody”, and “vanillin”, and identified several differential odorants between aged and unaged beer (Pang et al., 2025). A subsequent, broader metabolomic study further revealed that barrel aging induces substantial variation in volatile, semi-volatile, and non-volatile compounds in lager beer (Shi et al., 2026). However, these valuable insights are primarily derived from comparisons between discrete endpoints (e.g., unaged vs. aged beer), offering a static rather than a dynamic perspective. A systematic, time-resolved understanding of the simultaneous evolution of sensory attributes, physicochemical properties, and the full volatile metabolome throughout extended maturation is still lacking.

To address this gap, the present study integrates three analytical dimensions—quantitative descriptive sensory analysis, physicochemical profiling, volatile metabolomics—over a 300-day barrel aging period. The specific objectives were to: 1) characterize the dynamic changes of sensory attributes and physicochemical parameters; 2) map the time-course changes in the global volatile metabolome, identifying significantly differential metabolites and analyzing class-specific evolution patterns for key flavor-active compounds (e.g., esters, aromatic compounds, heterocyclic compounds); and 3) employ Mantel tests to statistically evaluate the correlations between aging time and both physicochemical/volatile compound data, and between quantitative sensory descriptors and flavor-active volatiles. This integrated approach aims to identify robust aging biomarkers and establish quantitative chemical-sensory links, providing a comprehensive framework for understanding and ultimately controlling flavor development in barrel-aged beers.

## 2 Materials and methods

### 2.1 Sample collection

Beer samples were provided by Tsingtao Brewery Co., Ltd. (Qingdao, Shandong Province, China). The brewing process was detailed in our previous patent (Huang et al., 2023). All samples were produced from a single whole wheat lager wort and a single fermentation batch, yielding a strong lager beer with an ethanol concentration of 10–12% (*v*/v). For barrel aging, this base beer was divided into multiple American bourbon barrels and aged under controlled conditions (0–5 °C; 30–50% relative humidity) for up to 300 days. Sampling was performed at five time points (0, 60, 100, 180, and 300 days). At each time point, three independent barrels were sampled as biological replicates, resulting in a total of 15 samples. A quality control (QC) sample was prepared by pooling aliquots from all samples to monitor analytical performance. All samples were immediately flash-frozen in liquid nitrogen and stored at −80 °C until analysis.

### 2.2 Sensory evaluation

Sensory evaluation was conducted using quantitative descriptive analysis (QDA) based on our before results and methodology with some modifications (Pang et al., 2025). The panel consisted of 10 evaluation experts (35–50 years old) from the Tsingtao Brewery Company. During preliminary tests for barrel-aged beer samples, six odor notes, namely “caramel”, “wine-like”, “woody”, “vanillin”, “sweet”, and “coconut” were chosen by the sensory panel as the most representative characteristic odor attributes in the beer sample. The “wine-like” note refers to olfactory characteristics reminiscent of aged wine or sherry, including fruity-estery and subtle oxidized nuances. Each odor attribute was defined using a reference standard (Table S1) presented at 10 times its threshold concentration. Intensity was expressed on a 10-point scale ranging from 1 to 10 (1 indicating that the attribute was “not perceived” and 10 indicating “extremely strong”). This sensory study did not require ethical approval because it involved only the tasting of commercially available beer with no health or safety risks, and no sensitive personal data were collected. All panelists provided written informed consent before participation. The study was conducted in accordance with the ethical standards of the Declaration of Helsinki, and appropriate protocols were followed to protect the rights and privacy of all participants.

### 2.3 GC–MS analysis

Volatile compound analysis was performed using headspace solid-phase microextraction coupled with gas chromatography–Q-Exactive Orbitrap mass spectrometry (HS-SPME-GC–MS; Thermo Scientific, Germany). A TriPlus RSH autosampler (Thermo Scientific) was employed for automated sample introduction. Chromatographic separation was carried out on a TRACE 1310 gas chromatograph (Thermo Scientific) equipped with a TG-5SilMS capillary column (25 m × 0.25 mm × 0.25 μm; TraceGOLD, Thermo Scientific).

For sample preparation, 5 mL of beer was combined with 2.0 g NaCl in a 20 mL headspace glass vial. After vortex mixing (500 rpm, 5 min, 50 °C), volatiles were extracted using a DVB/CAR/PDMS-coated SPME fiber (50/30 μm × 1 cm; Supelco, PA) for 60 min at 50 °C. Thermal desorption of the fiber was performed in the GC injector at 250 °C for 2 min (splitless mode). Helium carrier gas was maintained at a constant flow rate of 1.2 mL/min. The oven temperature program consisted of: 40 °C (hold 10 min) → 200 °C at 3 °C/min (hold 30 min) → 250 °C at 30 °C/min (hold 5 min).

Mass spectrometric detection was conducted in electron ionization (EI) mode with full-scan acquisition (*m*/*z* 45–400) at 70 eV ionization energy. Orbitrap mass analyzer was operated at 60,000 resolution (m/z 200). Both ion source and transfer line temperatures were set to 250 °C.

### 2.4 Determination of physicochemical parameters

The physicochemical parameters of the beer samples—including color, alcohol content, total acid, pH, total polyphenol, and bitterness—were analyzed as follows. The pH was measured using a pH meter (Mettler Toledo-Seven Compact, Switzerland). Alcohol content was determined directly by selective near-infrared (NIR) absorption using an Alcolyzer Beer ME (Anton Paar GmbH, Graz, Austria). Total acid was quantified by acid–base titration according to the Chinese National Standard (GB/T 12456–2021). Beer color was assessed following the ASBC Beer-10 method. Absorbance was measured at 430 nm and 700 nm, and color units were calculated using the specified conversion factor (× 22).

Total polyphenol content was determined based on the formation of a red complex with an iron reagent, measured at 600 nm. Briefly, 10 mL of beer was mixed with 8 mL of sodium carboxymethyl cellulose/EDTA solution (1% CMC / 0.2% EDTA, *w*/*v*) and 0.5 mL of ferric ammonium citrate solution (Fe(NH₄)₃(C₆H₅O₇)₂, 35% *w*/w, 16% iron) in a 25 mL volumetric flask. After adding 0.5 mL of ammonia solution (1:2, *v*/v) and diluting to volume, the mixture stood for 10 min before measurement. A blank was prepared identically but without ferric ammonium citrate. Total polyphenol (mg/L) was calculated as:

$$\text{Total polyphenol}=\left(A_{S}–A_{B}\right)\times 820$$

where A_S_ and A_B_ are the absorbances of the sample and blank at 600 nm, respectively, and 820 is an empirical factor.

Bitterness (in International Bitterness Units, IBU) was measured according to a modified spectrophotometric method at 275 nm (Wang et al., 2025). A degassed beer sample (10 mL) was mixed with 1 mL of 3 M HCl and 20 mL of isooctane, shaken mechanically at 150 rpm for 15 min, and centrifuged at 3500 rpm for 15 min. The supernatant was measured in a 10 mm quartz cuvette using isooctane as a blank. Bitterness was calculated as:

$$\text{Bitterness}\;\left(\mathrm{IBU}\right)=\left(A_{S}–A_{B}\right)\times 50$$

where A_S_ and A_B_ are the absorbances of the sample and blank at 275 nm, and 50 is the empirical conversion factor.

### 2.5 Data analysis

To investigate the dynamic changes in volatile metabolites during barrel aging, multivariate statistical analyses were performed using three complementary approaches: principal component analysis (PCA), orthogonal partial least squares-discriminant analysis (OPLS-DA), and hierarchical cluster analysis (HCA) with heatmap visualization. All analyses were conducted in MetaboAnalyst 6.0 (https://www.metaboanalyst.ca/). Significantly different metabolites were identified based on a variable importance in projection (VIP) score > 1 from OPLS-DA, combined with a Student's *t*-test threshold of *p* < 0.05 and a fold change ≥ 2 or ≤ 0.5.

For visualization, the distribution of metabolite classes was plotted as pie charts using OriginPro 2025 (OriginLab Corporation). Pearson correlation coefficients were calculated using R-language for Mantel test correlation analysis to examine relationships among key flavor characterization, sensory attributes, and physicochemical parameters.

## 3 Results and discussion

### 3.1 Dynamic changes in physicochemical factors

Physicochemical parameters were monitored throughout the barrel-aging process (Table 1). A steady increase in beer color was observed during aging. This color deepening can be attributed to the extraction of ellagitannins from oak wood (Vivas et al., 2020) as well as to the oxidative polymerization of polyphenols, which form colored complexes over time (Wannenmacher et al., 2018). Concomitantly, total polyphenol content decreased from 470.70 ± 5.6 mg/L to 373.10 ± 44.65 mg/L. This decrease likely results from multiple pathways: oxidative degradation of polyphenols (Vanderhaegen et al., 2006), polymerization and precipitation of high-molecular-weight polyphenols, and adsorption onto the porous wood matrix (Wannenmacher et al., 2018). The reduction in total polyphenols was accompanied by a decrease in beer bitterness. During wort boiling, hop α-acids are isomerized into isoα-acids, which are the main bitter compounds in beer (Medina et al., 2023). During extended barrel aging, trans-isoα-acids undergo proton-catalyzed cyclization to form tri- and tetracyclic degradation products with much lower bitterness intensity, explaining the observed bitterness decline (Karabín et al., 2014). This pattern is consistent with findings in IPA beers (Moura-Nunes et al., 2016).

**Table 1:** Changes in physicochemical properties during barrel-aging.

Aging Time (days)	Color	Total polyphenol (mg/L)	Bitterness (BU)	Total acid (mL/100 mL)	pH	Ethanol (%vol)
0	38.30 ± 0.50^a^	470.70 ± 5.6^a^	26.90 ± 0.6^a^	3.21 ± 0.00^a^	4.38 ± 0.01^a^	10.92 ± 0.20^a^
60	41.35 ± 0.21^b^	428.00 ± 12.18^b^	23.10 ± 0.49^b^	3.29 ± 0.02^b^	4.41 ± 0.01^a^	11.45 ± 0.31^a^
100	41.35 ± 0.35^b^	420.70 ± 5.80^b^	21.65 ± 1.20^b^	3.34 ± 0.00^c^	4.39 ± 0.01^a^	11.47 ± 0.16^a^
180	42.30 ± 0.42^b^	401.80 ± 2.26^c^	21.00 ± 0.57^b^	3.35 ± 0.00^d^	4.37 ± 0.01^a^	11.51 ± 0.11^a^
300	45.56 ± 0.85^c^	373.10 ± 44.65^d^	17.90 ± 0.57^c^	3.40 ± 0.02^e^	4.38 ± 0.02^a^	11.52 ± 0.43^a^

Values within each column followed by different letters indicate significant differences (p < 0.05).

Total acidity increased from 3.21 ± 0.00 mL/100 mL to 3.40 ± 0.02 mL/100 mL, while pH remained relatively stable. The buffering capacity of beer—derived from phosphates, proteins, and organic acids—explains why pH does not change substantially despite the accumulation of organic acids extracted from wood or generated by microbial activity (Li et al., 2016). Ethanol concentration increased non-significantly from 10.92 ± 0.20% to 11.52 ± 0.43% over the 300-day period. This modest rise is likely due to the extraction of residual ethanol from the Bourbon barrels, as whiskey trapped in the wood diffuses into the beer during extended contact.

### 3.2 Descriptive sensory analysis of beers

**Fig. 1:**
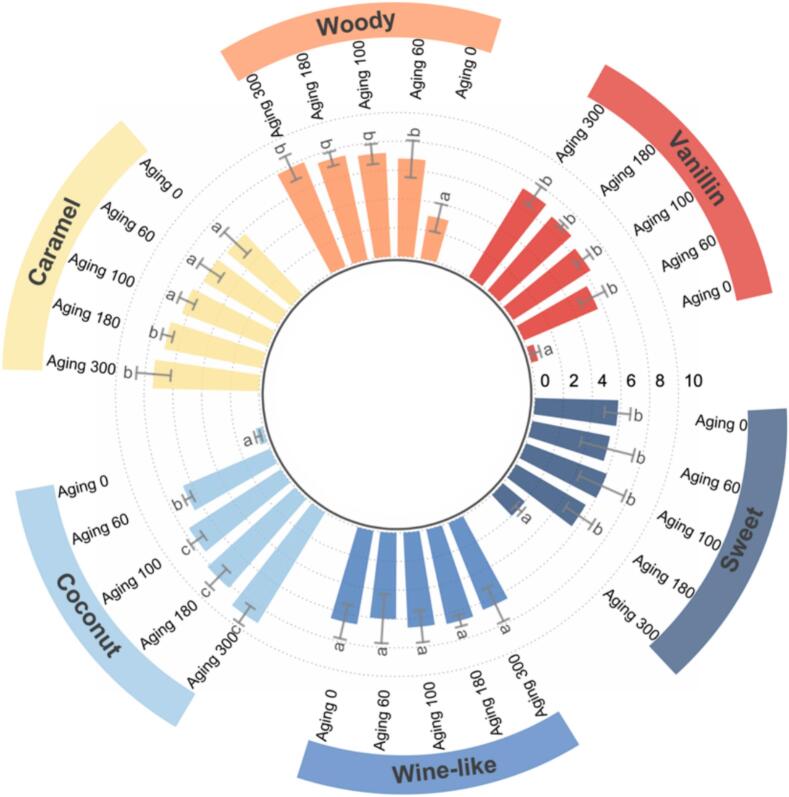
Comparative quantitative descriptive analysis (QDA) of aroma profile of beer samples (Values within each column followed by different letters indicate significant differences (p < 0.05)).

Six odor note attributes—caramel, wine-like, woody, vanillin, sweet, and coconut—were selected by the sensory panel through discussion and consensus as the most representative odor characteristics of the barrel-aged beer samples. Each attribute was evaluated on a 10-point scale by 10 panelists, and the results are presented as mean ± SD (*n* = 10) in Fig. 1. Significant changes in the odor profile were observed following barrel aging **(**Fig. 1**)**. Specifically, unaged beers exhibited a dominant wine-like note, accompanied by moderate caramel notes and slight nutty and hoppy nuances. After barrel aging, new odor notes emerged, including distinct coconut and vanillin notes, while woody and sweet characteristics were enhanced. In contrast, the wine-like note became less pronounced. Over extended aging, caramel, vanillin, coconut, and woody attributes showed progressive increases, with significant differences from day 0 to day 300 (*p* < 0.05), although differences between some intermediate time points were not statistically significant. Wine-like notes remained relatively stable without significant overall change between unaged and aged samples, while sweet notes showed no significant change throughout aging (*p* > 0.05). The emergence and intensification of coconut and vanillin notes can be attributed to the extraction of cis-oak-lactone and vanillin from the oak wood, as previously reported in barrel-aged beers and spirits (Niu et al., 2017; Pang et al., 2025). The enhancement of woody and caramel characteristics is consistent with the release of guaiacol and furanic compounds from toasted wood (Sterckx et al., 2012a). Overall, barrel aging contributed to a more pleasant, delicate, and harmonious odor profile. The correlation analysis between these sensory attributes and specific volatile compounds are further explored in Section 3.6.

### 3.3 Full mass spectrometric analysis of volatile metabolites

**Fig. 2:**
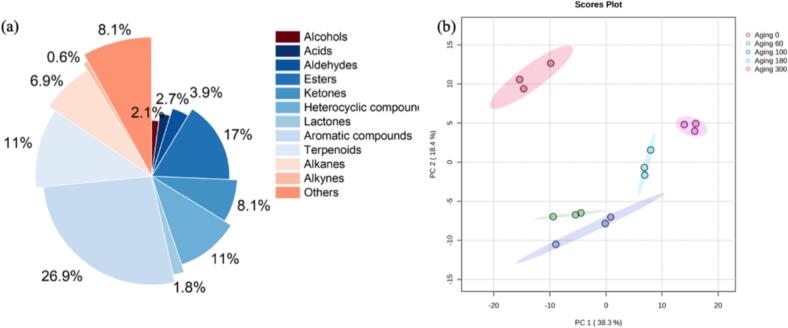
(a) Pie chart of the classification of all volatile compounds. (b) Scatter plot obtained from two extracted components of PCA analysis based on volatile components.

In this study, HS-SPME-GC–MS analysis was conducted to compare volatile compound profiles across five beer samples aged 0–300 days (0, 60, 100, 180, and 300 days). A total of 8331 mass spectrometric features were extracted, with 1298 volatile compounds detected above the intensity threshold (TIC >100,000). Volatile compounds were identified by aligning retention times, mass spectra similarity (>60%), and *m*/*z* ratios with reference standards and the NIST library. Subsequent identification confirmed 335 volatile compounds categorized into 12 classes: 7 alcohols, 9 acids, 13 aldehydes, 57 esters, 27 ketones, 37 heterocyclic compounds, 6 lactones, 90 aromatic compounds, 2 alkynes, 37 terpenoids, 23 alkanes, and 27 miscellaneous compounds **(**Fig. 2a**)**. Then, normalized peak areas were used for statistical analyses and quantitative comparisons of volatile compound abundances (e.g., increases/decreases). These compounds were subsequently subjected to multivariate statistical analysis. Principal component analysis (PCA) incorporating quality control (QC) samples demonstrated distinct clustering patterns **(Fig. S1a)**, with QC samples tightly aggregated within the 95% confidence interval, confirming experimental repeatability and data reliability.

To investigate temporal trends in volatile components during barrel aging, a PCA model was constructed using the 335 identified compounds **(**Fig. 2b**)**. The scatter plot revealed clear intergroup separation, indicating substantial compositional differences between aging stages. Specifically, unaged beer clustered in the upper left quadrant, 60–100-day samples occupied the lower left, while 180–300-day samples distributed to the lower right. This spatial progression suggests stepwise modifications in volatile metabolites during aging, with the extent of alteration increasing progressively with aging duration. The temporal trend became more pronounced in supervised OPLS-DA analysis **(Fig. S1b)**, where samples aligned sequentially from left to right with increasing aging duration. Collectively, these findings demonstrated that barrel aging induced significant modifications in volatile composition, with the extent of these alterations increasing progressively with aging duration (Tarko et al., 2023). The specific compounds contributing to these separations are further explored in Section 3.4, where pairwise OPLS-DA and statistical screening identify differential metabolites between consecutive time points.

### 3.4 Differential volatile compounds screening

To elucidate the impact of aging on volatile compounds at the various stage of barrel-aged beer, key distinctions during aging process were explored. Specifically, orthogonal partial least squares-discriminant analysis (OPLS-DA) was performed as pairwise comparisons between consecutive time points: 60 days vs. 0 days, 100 days vs. 60 days, 180 days vs. 100 days, and 300 days vs. 180 days. Each comparison included six samples (three biological replicates per time point). Volatile compounds exhibiting significant differences were screened based on variable importance in projection (VIP) > 1, Student's *t*-test *p* < 0.05, and fold change ≥2 or ≤ 0.5.

**Fig. 3:**
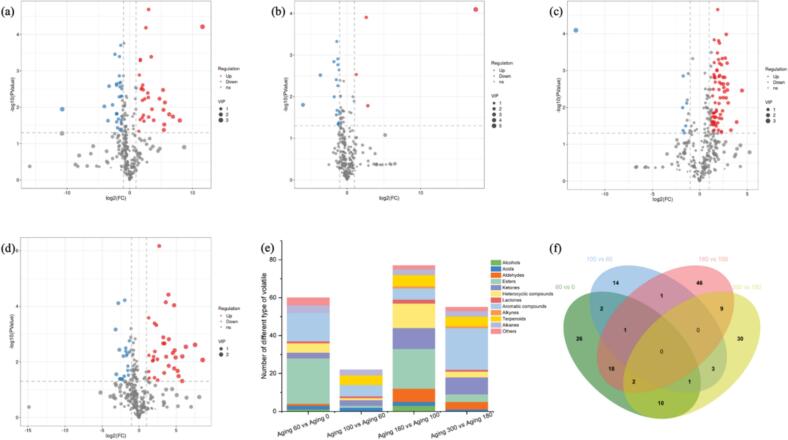
Differential volatile metabolites present in barrel-aged beer. (a–d) The volcano plot of the differential volatile metabolites of aging 60 days vs. aging 0 days, aging 100 days vs. aging 60 days, aging 180 days vs. aging 100 days, and aging 300 days vs. aging 180 days. (e) The bar graph comparing the number and classification. (f) The Venn diagram.

The volcano plots reflected the information on differential metabolite up-regulation and down-regulation **(**Fig. 3a-d**)**. A total of 162 different substances were screened, out of which 60 differences were observed between 60 days aged beer and unaged beer (32 up, 28 down), 22 differences were recorded between 100 days aged beer and 60 days aged beer (4 up, 18 down), 76 differences between 180 days aged beer and 100 days aged beer (70 up, 6 down), 54 differences between 300 days aged beer and 180 days aged beer (34 up, 20 down). As shown in Fig. 3e, the number of differential substances were variable as the barrel-aging process, and the most abundant different substances noted in aging 180 vs. aging 100. In another word, the effect of barrel-aging on volatile compounds was unsteady as the aging time increased. Notably, esters, aromatic compounds, ketones and heterocyclic compounds were the main chemical categories that changed during the barrel-aging process.

Venn diagram exhibited the distinction and intersection of differential volatile compounds numerically during barrel-aging process **(**Fig. 3f**)**. None volatile compound was common in each comparison group. There were 26, 14, 46, and 30 unique differential volatile compounds between aging 60 vs. aging 0, aging 100 vs. aging 60, aging 180 vs. aging 100, and aging 300 vs. aging 180, respectively. The 46 unique volatile compounds to aging 180 vs. aging 100 accounted for 28.2% of the total differential substances, which indicating that the 180 days' barrel-aging was a key step for the barrel-aged beer taste.

### 3.5 Dynamic changes in different volatile metabolites during barrel aging processing

**Fig. 4:**
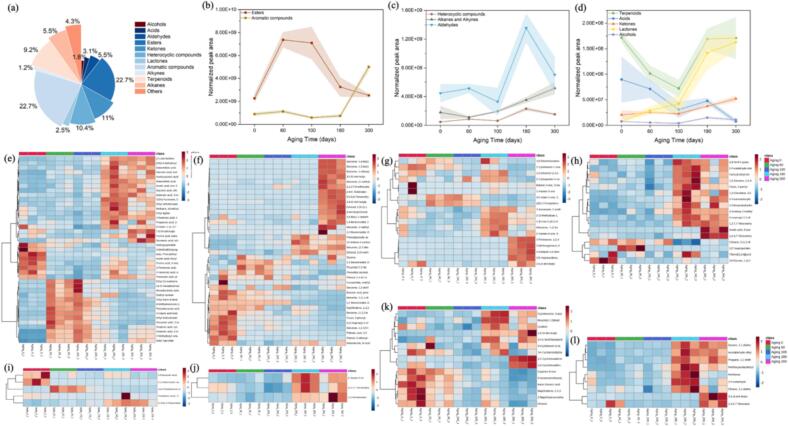
(a) Pie chart displaying the classification and distribution of 163 different volatile metabolites; (b-d) the dynamic changes of different volatile metabolites during barrel aging process; Heatmap of volatile components content including (e) esters and lactones; (f) aromatic compounds; (g) ketones; (h) heterocyclic compounds; (i) acids; (j) alcohols; (k) terpenoids; (l) aldehydes.

As depicted in Fig. 4a, 162 different volatile metabolites were assigned to 12 classes, including 37 esters, 37 aromatic compounds, 18 ketones, 16 heterocyclic compounds, 15 terpenoids, 9 aldehydes, 5 acids, 3 alcohols, 4 lactones, 9 alkanes, 2 alkynes, and 7 others. During the barrel-aging process, the dynamic changes of those chemical classes of volatile compounds reflected by normalized peak area has been displayed in Fig. 4b-d. Heat maps analysis **(**Fig. 4e-l**)** elucidated specific variation trends among these different metabolites throughout the barrel aging processing.

#### 3.5.1 Ester

Esters are crucial for beer's flavor profile, contributing desirable sweet and fruity notes (Chen et al., 2025; Pirrone et al., 2025). Our analysis demonstrated a dynamic evolution of ester compounds during the 300-day oak aging process **(**Fig. 4b**)**. The total ester content followed a non-linear trend: it increased rapidly within the first 60 days, stabilized around day 100, and then decreased sharply by day 180. By the end of aging, the overall ester content returned to levels comparable to those in unaged beer.

Based on their distinct aging behavior, the esters were classified into four categories. The first group, consisting of esters with high initial concentrations, showed an immediate increase after barrel entry, maintained elevated levels until day 100, and then declined. This group included 8-methylnonanoic acid, ethyl ester (CAS: 905589–94-8), the most abundant ester (≥53.64% of peak area) with undocumented flavor properties; ethyl trans-4-decenoate (CAS: 76649–16-6), contributing ≥23.26% of peak area with green, fruity, waxy, and cognac-like notes, previously identified in fermented products (Li et al., 2017; Liu et al., 2025); and ethyl tridecanoate (CAS: 28267–29-0), known for its baked apple notes and recognized as a flavor marker in maize spirits (Weiss et al., 2023). These ethyl esters, already present at high levels in unaged beer, likely increased through esterification between corresponding fatty acids and ethanol, reached a plateau indicating esterification-hydrolysis equilibrium, and subsequently decreased due to adsorption by oak barrel.

The second group of esters remained relatively stable in the initial stages but increased substantially later in aging. This category includes butanedioic acid, diethyl ester (CAS: 123–25-1; floral/fruity attributes), which increased after 180 days, and nonanoic acid, ethyl ester (CAS: 123–29-5; rosaceous/fruity notes), which rose by day 300. The delayed increase suggests slower esterification kinetics, possibly due to lower precursor fatty acid concentrations compared to the first group. This observation aligns with reports of nonanoic acid, ethyl ester enrichment in oak-aged wines (Mislata et al., 2021; Pérez-Prieto et al., 2003), supporting the notion of ongoing esterification during maturation.

The third group demonstrated a gradual, continuous increase throughout aging, including compounds such as octanoic acid, 3-methylbutyl ester (CAS: 2035-99-6; coconut/sweet notes), and 2-methylbutyl octanoate (CAS: 67121–39-5). Their steady accumulation may result from esterification between fatty acids and branched-chain alcohols (2-methylbutanol or 3-methylbutanol) present in beer. However, the considerably lower concentrations of these alcohols compared to ethanol likely account for the slower esterification rate (Callejo et al., 2019).

The fourth group exhibited a U-shaped concentration profile, decreasing initially before recovering in later stages. Notable flavor-active esters in this category include ethyl 4-(ethyloxy)-2-oxobut-3-enoate (CAS: 76240–19-2), butanoic acid, 3-methyl-, ethyl ester (CAS: 108–64-5; fruity, sweet, apple, pineapple), acetic acid, hexyl ester (CAS: 142–92-7; fruity, green, apple, banana, sweet), pentanoic acid, propyl ester (CAS: 141–06-0; fruity, pineapple, metallic), and 6-octen-1-ol, 3,7-dimethyl-, acetate (CAS: 150–84-5; floral, rose, citrus, woody, tropical fruit). Other esters like 2-pentenoic acid, 4,4-dimethyl-, methyl ester (CAS: 16812–85-4) showed similar trends. The initial decrease may reflect adsorption by oak surpassing esterification rates at low concentrations, while subsequent recovery suggests a shift in the esterification-hydrolysis equilibrium toward net synthesis as concentrations decline.

In summary, the oak aging process drives complex, time-dependent modifications in the beer's ester profile, directly influencing its sensory characteristics. The four distinct evolution patterns observed-initial rise and decline, delayed increase, gradual accumulation, and U-shaped recovery-reflect the dynamic interplay between chemical reactions (esterification and hydrolysis) and physical processes (oak adsorption). These transformations lead to substantial fluctuations in fruity, sweet, and floral notes throughout maturation, with the final ester composition approaching pre-aging levels after 300 days.

#### 3.5.2 Aromatic compounds

Aromatic compounds significantly influence the organoleptic properties of beer, particularly by modulating mouthfeel, astringency, and after-bitterness (Gil et al., 2003). They contribute diverse odor notes, including floral, sweet, and smoky characters (Chen et al., 2025). During barrel aging, the total level of these compounds remained relatively stable in the initial phase but increased rapidly after 180 days. This trend was predominantly driven by the behavior of 2,4-Di-tert-butylphenol (CAS: 96–76-4), which consistently accounted for over 43.90% of the total peak area. Its concentration showed little variation in the early aging stage but rose sharply after 180 days. This compound, characterized by phenolic and musty odors, is a known yeast metabolite (Williams et al., 2023) and has been previously detected in blonde beer (Zanella et al., 2021). Another notable compound, (2,2-diethoxyethyl) benzene, (CAS: 6314-97-2), imparts fresh, green, bluebell, almond, and sweet notes. Its concentration increased gradually throughout the aging process. This compound has been identified in various barrel-aged spirits such as apple brandy, sherry, and wine (Api et al., 2022), suggesting its origin is likely the oak wood itself, with a slow leaching into the beer over time. The concentration of 1,3-bis(1,1-dimethylethyl) benzene (CAS: 1014-60-4) was low and stable before 180 days, after which it increased. While its specific aroma profile remains unreported, it has been detected in numerous fermented foods. Literature indicates that 1,3-bis(1,1-dimethylethyl) benzene is primarily biosynthesized by fungi (e.g., *Aspergillus* spp., Ganoderma lucidum) and is found in products like rice wine and black tea (Ripari et al., 2016; Tietel & Masaphy, 2022). Its presence in aged beer may therefore originate from fungi colonizing the interior surfaces of the oak barrels.

In summary, the evolution of key aromatic compounds during barrel aging follows distinct temporal patterns, significantly shaping the beer's final sensory profile. The overall level remained stable initially before increasing after 180 days, a pattern primarily dictated by the yeast-derived compound 2,4-di-tert-butylphenol. Concurrently, the gradual rise of phenylacetaldehyde diethyl acetal indicates its extraction from oak wood, while the late-stage increase of 1,3-bis(1,1-dimethylethyl) benzene suggests a potential fungal origin. These evolving compounds collectively enrich the beer's sensory complexity by introducing phenolic, sweet, green, and microbial-metabolic nuances throughout maturation.

#### 3.5.3 Heterocyclic compounds

Heterocyclic compounds are important contributors to the roasted, nutty, floral, and fruity flavors of beer (Yue et al., 2023). During barrel aging, their total concentration exhibited a sigmoidal evolution: levels remained relatively stable initially, increased between 100 and 180 days, and then stabilized again. This trend was largely defined by two major constituents: 1,3-Dioxane, 2,4-dimethyl- (CAS: 766–20-1; >24.08% of peak area) and 3-Acetoxy-2-methyl-pyran-4-one (>4.6%). The former has been rarely reported; its presence in plants (Thakur et al., 2024) suggests a potential wood origin. The latter, a pyran derivative, has not been well-documented in the literature.

A similar sigmoidal profile was observed for several other heterocyclic compounds, including Furfuryl ethyl ether (CAS: 6270-56-0; sweet, spicy, nutty, coffee), 2-Furaldehyde diethyl acetal (CAS: 13529–27-6; fruity, earthy, mushroom), 1,3-Dioxolane, 4,5-dimethyl-2-pentadecyl- (CAS: 56599–61-2), and Ethyl 2-furoate (CAS: 614–99-3; fruity, floral, burnt). Their origins appear diverse: Furfuryl ethyl ether likely forms in beer from furfuryl acetate via furfuryl alcohol (Spillman et al., 1998); 2-Furaldehyde diethyl acetal is widespread in foods and beverages (Sethupathy et al., 2015); and Ethyl 2-furoate may be introduced during oak aging, as shown in studies of other spirits (Sethupathy et al., 2015). Notably, barrel toasting plays a critical role in generating these compounds, significantly enhancing their diversity and abundance (Yue et al., 2023).

In summary, heterocyclic compounds exhibited a sigmoidal concentration profile during aging, shaped primarily by two major constituents. Their increase correlates with both wood-derived inputs and barrel toasting, collectively enhancing roasted, nutty, and fruity notes in the final beer.

#### 3.5.4 Terpenoids

Terpenoids constitute a class of compounds significantly associated with the floral and “hoppy” sensory character of beer (Williams et al., 2023). During barrel aging, the overall terpenoid content exhibited a trend of initial decline followed by an increase after approximately 100 days. This pattern was primarily driven by the behavior of linalool (CAS: 78–70-6), which imparts fresh floral and citrus notes. The observed increase in linalool during later stages of barrel aging aligns with findings reported in red wine studies (Pichler et al., 2025). While linalool in beer originates from hops and is generally considered susceptible to oxidative degradation during storage (Silva Ferreira & Collin, 2021), its concentration rise in the present experiment may be attributed to its extraction from oak wood (Pichler et al., 2025) or to its non-enzymatic formation from precursors such as nerol or geraniol during aging (Khvalbota et al., 2021).

In contrast to linalool, several other aroma-active terpenoids showed a gradual declining trend throughout the aging process. Examples include guaiyl acetate (CAS: 134–28-1; tea rose, woody, spicy, green, fatty), trans-geranic acid methyl ester (CAS: 1189-09-9; waxy, green, fruity, floral), valencene (CAS: 4630-07-3; citrus, fruity, peel, woody), and γ-eudesmol (CAS: 1209-71-8; waxy, sweet). This general decrease can be explained by the well-documented susceptibility of terpenes to oxidation during beer brewing, storage, and aging (Medina et al., 2023), coupled with a lack of significant replenishment via extraction from the oak wood.

In summary, terpenoids displayed divergent behaviors during barrel aging. While linalool increased after an initial decline, most other aroma-active terpenoids decreased gradually. These dynamics collectively modulate the evolving floral and hoppy character of barrel-aged beer.

#### 3.5.5 Others

Other compounds also exhibited defined trends during oak aging. Lactones, for instance, showed a progressive increase, as illustrated by (*Z*)-oak-lactone (CAS: 55013–32-6) with its coconut and vanilla notes. These cyclic carboxylic esters, which form mainly during maceration and fermentation, can be extracted from wood into beer during barrel aging and are considered indicators of oak maturation. A similar accumulation of lactones has been reported during the oak aging of Cabernet Sauvignon wine (Pichler et al., 2025).

Aldehydes displayed minimal fluctuation in the early stages of aging but increased substantially after 100 days. This rise may originate from multiple pathways, including linoleic acid autoxidation, Strecker degradation of amino acids, oxidation of higher alcohols, and Maillard reactions (Aguiar et al., 2022). This observed trend aligns with findings reported by Wyler et al. (2015) A representative aldehyde, acetaldehyde diethyl acetal (CAS: 105–57-7; nutty, earthy, vegetable), followed a similar pattern: its concentration varied initially before increasing after 100 days, a progression that may be attributed to synthesis becoming favored when the initial concentration is low (Madrera et al., 2003).

### 3.6 Correlation analysis of chemical changes with sensory evolution and aging durations

Based on the findings outlined above, the volatile composition of beer undergoes systematic transformation throughout the 300-day barrel-aging process. To statistically link these chemical changes with sensory evolution and aging duration, Mantel tests were conducted. These analyses evaluated correlations between sensory attributes and key aroma metabolites, as well as between aging time and physicochemical or volatile metabolite profiles, aiming to identify potential biomarkers of aging progression. As shown in Fig. 6, line thickness corresponds to the Mantel's correlation coefficient (r), while color indicates statistical significance (p); thick blue and green lines denote strong correlations. Mantel analysis confirmed that aging time is significantly associated with both physicochemical properties and aroma-related characteristics.

**Fig. 5:**
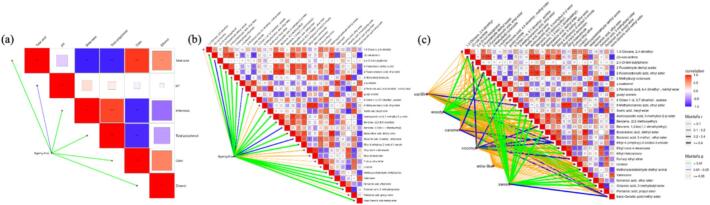
Combined graph of Mantel test and Spearman correlation heatmap. (a) Correlation analysis between aging time and physicochemical factors, (b) Correlation analysis between aging time and selected key differential aroma-related compounds, (c) Correlation analysis between sensory characteristics and key differential aroma-related compounds.

Fig. 5a reveals that four of the six physicochemical properties measured—total acidity, bitterness, total polyphenols, and color—were highly correlated (|r| > 0.8, *p* < 0.01) with aging time. These correlations reflect the integrated effects of extraction, oxidation, and condensation reactions during maturation. Notably, the observed decrease in bitterness and increase in smoothness noted during sensory evaluation align with these physicochemical trends over extended aging.

Regarding volatile metabolites **(**Fig. 5b**)**, among the 28 aroma features analyzed, three compounds showed a strong correlation (|r| > 0.8, p < 0.01) with aging time: (*Z*)-oak-lactone, 2-furancarboxylic acid ethyl ester (ethyl 2-furoate), and (2,2-diethoxyethyl) benzene. An additional four compounds exhibited moderate correlation (0.7 < |r| < 0.8): acetoxyacetic acid 3-methylbut-2-yl ester, butanedioic acid diethyl ester, methoxyacetaldehyde diethyl acetal, and trans-geranic acid methyl ester. The consistent, time-dependent accumulation of (Z)-oak-lactone, ethyl 2-furoate, and (2,2-diethoxyethyl) benzene supports their potential as chemical biomarkers for monitoring barrel-aging progression.

To further elucidate how specific notes relate to sensory perception, Mantel tests were also performed between sensory attributes and aroma-active volatile compounds **(**Fig. 5c**)**. The results indicated that several esters—including acetic acid hexyl ester, octanoic acid 3-methylbutyl ester, nonanoic acid ethyl ester, and butanedioic acid diethyl ester—were significantly positively correlated with sweet note (*r* > 0.717, *p* < 0.005). Meanwhile, (*Z*)-oak-lactone showed strong associations with both sweet (*r* = 0.897, p < 0.005) and woody (*r* = 0.894, p < 0.005) notes.

These integrated statistical analyses substantiate that barrel aging drives coherent changes in both the chemical composition and sensory profile of beer. Key wood-derived and fermentation-derived volatiles not only correlate strongly with aging time but also link directly to specific sensory attributes, providing a quantitative foundation for understanding and potentially steering flavor development during maturation.

## 4 Conclusion

This 300-day integrated study provides a comprehensive, time-resolved characterization of the chemical and sensory transformations occurring in beer during oak barrel aging. By combining quantitative sensory analysis, physicochemical monitoring, and volatile metabolomics, we systematically delineated the dynamic evolution of flavor-active compounds and their corresponding sensory outcomes.

The findings demonstrate that barrel aging drives class-specific evolution patterns governed by distinct physicochemical mechanisms. Esters exhibited four evolution trajectories (rapid rise/decline, delayed increase, gradual accumulation, U-shaped recovery) shaped by the interplay of esterification, hydrolysis, and adsorption; aromatic compounds showed delayed increases linked to yeast metabolism and wood extraction; heterocyclic compounds followed sigmoidal accumulation associated with wood-derived inputs; and terpenoids displayed divergent behaviors reflecting their differential susceptibility to oxidation and wood contribution.

Through Mantel correlation analysis, three wood-derived volatiles—(Z)-oak-lactone, ethyl 2-furoate, and (2,2-diethoxyethyl) benzene were identified as robust biomarkers strongly correlated with aging time (|r| > 0.8). Notably, (Z)-oak-lactone was quantitatively linked to both “woody” and “sweet” sensory attributes (*r* > 0.89), establishing a direct bridge between chemical composition and perceived flavor. These biomarkers offer practical tools for objectively monitoring aging progression and predicting flavor outcomes.

Beyond descriptive profiling, this work establishes a predictive chemical-sensory framework that advances fundamental understanding of barrel aging chemistry. These findings offer practical tools for brewers to optimize aging protocols and tailor flavor profiles. Future work should validate the identified biomarkers across diverse beer styles and barrel types.

## CRediT authorship contribution statement

**Yaqi Shi:** Writing – review & editing, Writing – original draft, Methodology, Investigation, Data curation, Conceptualization. **Junhong Yu:** Supervision, Resources, Conceptualization. **Lei Xing:** Supervision, Resources, Methodology. **Shumin Hu:** Supervision, Resources. **Hualei Chen:** Writing – original draft, Methodology, Investigation, Data curation. **Peng Yan:** Resources, Investigation, Data curation. **Shuping Shao:** Project administration, Data curation. **Haixin Liu:** Methodology, Investigation. **Yajie Tang:** Supervision. **Zhaoxia Yang:** Writing – review & editing, Supervision, Funding acquisition, Conceptualization. **Hua Yin:** Supervision, Resources, Funding acquisition.

## Declaration of competing interest

The authors declare that they have no known competing financial interests or personal relationships that could have appeared to influence the work reported in this paper.

## Data Availability

Data will be made available on request.
