# Supplementary material for: Dynamic changes of flavor characterization of lager beer during barrel aging process based on untargeted volatilomics
Source: Food Chem X. 2026 Apr 22;36:103901. doi: 10.1016/j.fochx.2026.103901 (PMC13134020; doi:10.1016/j.fochx.2026.103901)
Supplement: Supplementary file 1 — Supplementary material [file mmc1.docx]

Supplementary Material for

**Dynamic changes of flavor characterization of lager beer during barrel ageing process based on untargeted volatilomics**

**Index**

Table S1 The reference standards used for sensory analysis (Pang et al., 2025)

Fig. S1 (a) Scatterplot obtained from two extracted components of PCA analysis based on volatile components. (b) Score plot of OPLS-DA (R^2^X=0.505, R^2^Y=0.997, Q^2^=0.931)

**Table S1** The reference standards used for sensory analysis (Pang et al., 2025)

| **Odor note** | **Reference standard** | **CAS** | **Concentration/ppm** |
| --- | --- | --- | --- |
| caramel | maltol | 118-71-8 | 50 |
| wine-like | wine lactone | 182699-77-0 | 0.0001 |
| woody | guaiacol | 90-05-1 | 0.25 |
| vanillin | vanillin | 121-33-5 | 0.53 |
| coconut | cis-oaklactone | 55013-32-6 | 0.20 |
| sweet | phenethyl alcohol | 60-12-8 | 10 |

The standard for reference purposes is 10 times the threshold and present to the panel in 125 mL Teflon sniff bottles (Nalge Nunc International, Rochester, NY, U.S.A.).

**Reference:**

Pang, X., Yin, H., Li, J., Shi, Y., & Yang, Z. (2025). Molecular insights into the contribution of oak barrel aging to the aroma of beer with high alcohol content using SAFE-GC-O/AEDA and OAV calculation. *Food Chem, 491*, 145329. https://doi.org/10.1016/j.foodchem.2025.145329.


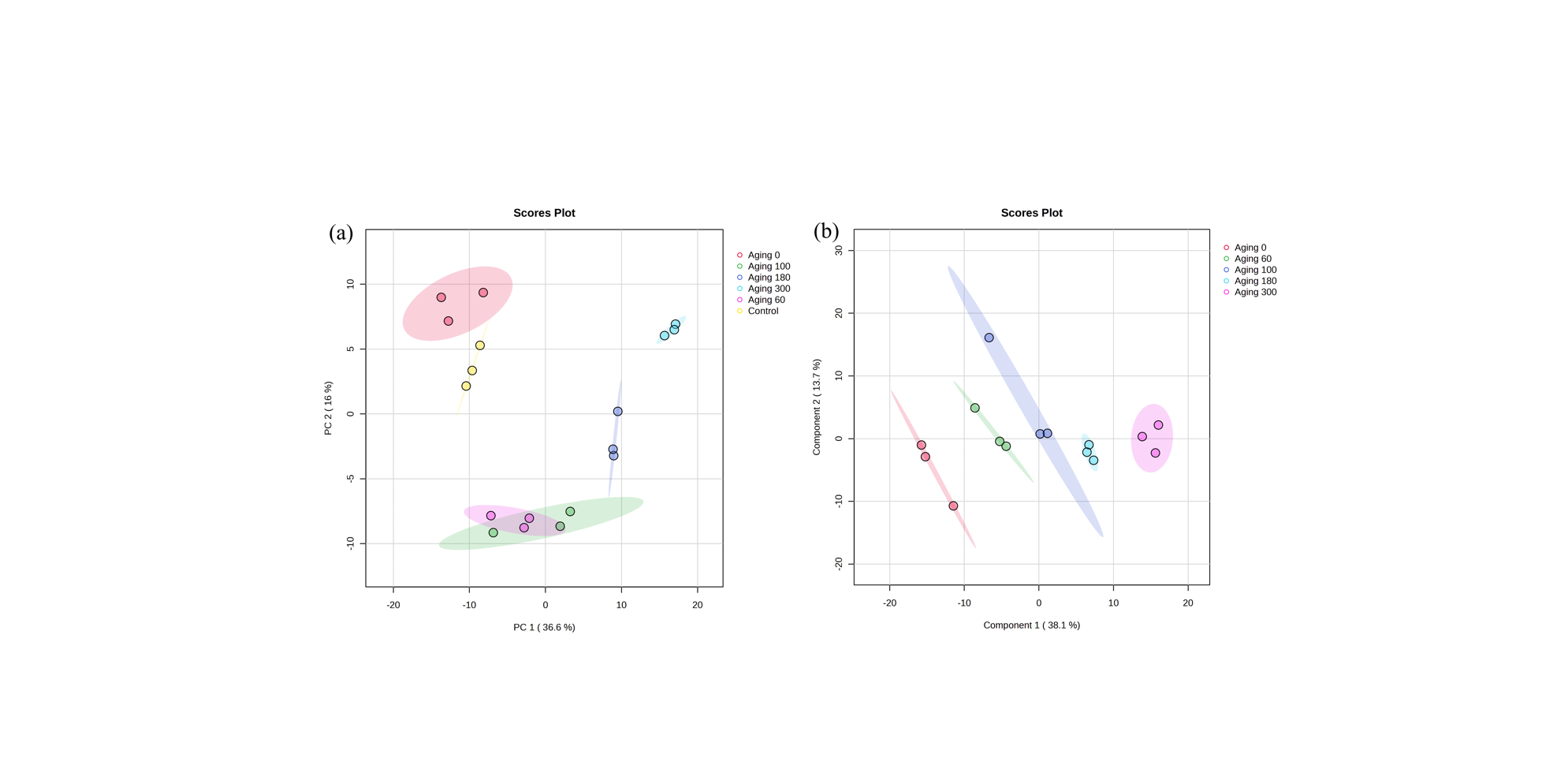


**Fig.S1** (a) Scatterplot obtained from two extracted components of PCA analysis based on volatile components. (b) Score plot of OPLS-DA (R^2^X=0.505, R^2^Y=0.997, Q^2^=0.931)
